# Control of *Fusarium graminearum* in Wheat With Mustard-Based Botanicals: From *in vitro* to *in planta*

**DOI:** 10.3389/fmicb.2020.01595

**Published:** 2020-07-21

**Authors:** Dimitrios Drakopoulos, Giuseppe Meca, Raquel Torrijos, Anja Marty, Andreas Kägi, Eveline Jenny, Hans-Rudolf Forrer, Johan Six, Susanne Vogelgsang

**Affiliations:** ^1^Ecological Plant Protection in Arable Crops, Plant Protection, Agroscope, Zurich, Switzerland; ^2^Sustainable Agroecosystems, Institute of Agricultural Sciences, ETH Zürich, Zurich, Switzerland; ^3^Food Chemistry and Toxicology, Faculty of Pharmacy, University of Valencia, Valencia, Spain

**Keywords:** Fusarium head blight, antifungal botanical, isothiocyanate, phenolic acid, mycotoxin, conidia, ascospores, wheat

## Abstract

*Fusarium graminearum* is a phytopathogenic fungus that causes Fusarium head blight in small-grain cereals, such as wheat, with significant yield reductions. Moreover, it contaminates the cereal grains with health-threatening mycotoxins, such as deoxynivalenol (DON), jeopardizing food and feed safety. Plant-based biopesticides, i.e. botanicals, have recently gained increased interest in crop protection as alternatives to synthetic chemical products. The main objective of this study was to test the control efficacy of botanicals based on white or Indian/Oriental mustard seed flours (Tillecur – Ti, Pure Yellow Mustard – PYM, Pure Oriental Mustard – POM, Oriental Mustard Bran – OMB) on *F. graminearum* infection and mycotoxin accumulation in wheat grain. Botanicals at 2% concentration showed a higher efficacy in inhibiting mycelium growth *in vitro* compared with a prothioconazole fungicide (F). In the growth chamber experiment under controlled conditions, the spraying agents reduced DON content in grain in the following order: F = Ti = PYM > POM > OMB. The antifungal activity of the botanicals may be attributed to their bioactive matrices containing isothiocyanates (ITCs) and phenolic acids. Allyl ITC was detected in POM and OMB at 8.38 and 4.48 mg g^–1^, while p-hydroxybenzyl ITC was found in Ti and PYM at 2.56 and 2.44 mg g^–1^, respectively. Considerable amounts of various phenolic acids were detected in all botanicals. Under field conditions, only the use of F significantly decreased *F. graminearum* infection and DON content in grain. An additional important finding of this study is that disease control was more difficult when infection was done with ascospores than conidia, which might have several potential implications considering that ascospores are more important in Fusarium head blight epidemics. Our results suggest that mustard-based botanicals are promising biopesticides for the control of Fusarium head blight in small-grain cereals, but for field applications, an appropriate formulation is necessary to stabilize and prolong the antifungal activity, especially against ascospores.

## Introduction

Several *Fusarium* species are phytopathogenic fungi, which mainly lead to Fusarium head blight (FHB) in small-grain cereals, such as wheat (*Triticum aestivum* L.), barley (*Hordeum vulgare* L.), triticale (× *Triticosecale* Wittmack), and oats (*Avena sativa* L.), as well as to Fusarium ear and stalk rot in maize (*Zea mays* L.). In most parts of the world, the most prevalent FHB causing species in wheat are within the *Fusarium graminearum* species complex (Osborne and Stein, 2007; Pasquali et al., 2016; Vogelgsang et al., 2019). Epidemics of FHB are frequently resulting in severe economic losses for cereal farmers due to significant reductions in grain yield and quality (Parry et al., 1995). Upon infection of the inflorescences, several *Fusarium* species produce health-threatening secondary metabolites, named mycotoxins, jeopardizing food and feed safety. The most toxicologically important mycotoxins produced by *F. graminearum* are deoxynivalenol (DON) and zearalenone (ZEN). The European Union and many other countries around the globe have established maximum levels in human food and guidance levels in animal feed (European Commission, 2006; Ferrigo et al., 2016). Thus, the management of FHB in wheat is crucial in order to minimize yield losses and reduce mycotoxin contamination to the lowest possible levels.

The control strategies of FHB in wheat can be categorized in prevention and intervention approaches. The prevention strategies include crop rotation with non-host plant species, management of remaining crop residues with tillage, and breeding for resistance. The intervention strategies mainly focus on protective measures of the cereal heads during anthesis (e.g. fungicides, biological control) and management strategies during or after harvest (e.g. grain processing) (Wegulo et al., 2015). Within the context of minimizing the negative impact of synthetic pesticides on the environment and the arising health-related public concerns, natural plant protection products have recently gained increased attention (Czaja et al., 2015). Botanicals are plant-based products that are frequently degrading faster than synthetic pesticides and therefore having potentially less negative impact on non-target organisms and less environmental risks (Regnault-Roger and Philogène, 2008). Botanicals have also been considered as “eco-chemical” and “bio-rational” products for the sustainable management of crop pests and diseases aiming to improve the safety of the producer, consumer, and the environment (Cavoski et al., 2011; Campos et al., 2016).

Mustard plants contain secondary metabolites, named glucosinolates (GSLs), which are hydrolyzed with the enzyme myrosinase into bioactive substances, i.e. isothiocyanates (ITCs). The effects of ITCs can be categorized into antimicrobial, herbicidal, and antioxidant. White mustard (*Sinapis alba* L.) and Indian/Oriental mustard (*Brassica juncea* L.) plants are commonly used as green manure in agricultural soils to suppress soil-borne pests and pathogens through biofumigation after biomass incorporation into the soil (Brown and Morra, 1997). Following another approach, botanical suspensions based on mustard seed flour could also be applied to crops for direct control of residue- and air-borne pathogens. In a previous study employing an array of *in vitro* bioassays, botanicals based on white mustard seed flour suppressed or fully inhibited the growth and development of several fungal structures of *F. graminearum* including mycelium, germination of conidia and ascospores, perithecia formation on maize stalks, and discharge of ascospores from mature perithecia produced on carrot agar (Drakopoulos et al., 2019). Apart from the GSLs, mustards also contain phenolic compounds. These compounds are utilized by plants for pigmentation, growth, reproduction as well as for resistance to pests and pathogens (Lattanzio et al., 2006). Phenolic acids, such as gallic, caffeic, cinnamic, benzoic, protocatechuic, and phenylacetic acids, were recorded to have antifungal activity (Teodoro et al., 2015). Ponts et al. (2011) showed that ferulic, coumaric, caffeic, syringic, and p-hydroxybenzoic acids inhibited the radial growth of four *F. graminearum* strains by 50%. Schöneberg et al. (2018a) demonstrated that ferulic acid substantially inhibited the mycelium growth of *F. graminearum*, *F. langsethiae* and *F. poae*, while p-hydroxybenzoic and vanillic acids had no effect.

The main objective of this study was to test the efficacy of mustard-based botanicals to reduce *F. graminearum* infection and mycotoxin accumulation in wheat. As a first step, the effects of botanicals at different concentrations were tested using a mycelium growth *in vitro* bioassay. Secondly, the efficacy of the botanicals was investigated under controlled conditions in the growth chamber using wheat plants that were artificially inoculated, with either conidia or ascospores. Subsequently, the efficacy of the botanicals was tested under field conditions in a wheat crop using a semi-artificial inoculation method with *F. graminearum*. Finally, the GSLs, ITCs, and phenolic acids present in the botanical powders were identified and quantified.

## Materials and Methods

### Fungal Strains and Spraying Agents

For the *in vitro* bioassay and the growth chamber experiment, the *F. graminearum* strain “2113” was used, while a mixture of three *F. graminearum* strains (i.e. “0410,” “2113,” “1145”; single-spore isolates from wheat grain in Switzerland; 15-acetyldeoxynivalenol genotypes) was used for the semi-artificial inoculation method in the field experiment. The botanical powders Tillecur^®^ (Ti; BIOFA, Germany), Pure Yellow Mustard (PYM; product code 106), Pure Oriental Mustard (POM; product code 107), and Oriental Mustard Bran (OMB; product code 403) were used for all experiments. PYM, POM, and OMB were provided by G. S. Dunn (Dry Mustard Millers, Canada). Ti and PYM are based on seed flour from white mustard, whereas POM is based on seed flour from Indian mustard. OMB is based on the seed husks from Indian mustard. The husks are commonly removed during the milling process and therefore are usually discarded. The synthetic fungicide (F) Proline^®^ (active ingredient: prothioconazole; Bayer AG Crop Science, Switzerland), which is commonly used by cereal farmers to control FHB, was included as a comparison in all experiments.

### *In vitro* Bioassay – Mycelium Growth

All botanicals were tested at 1 and 2%, while F was used at the recommended concentration (0.16%). The methodological procedure for this bioassay is provided in Drakopoulos et al. (2019). In brief, mycelial plugs of 0.5 cm diameter were cut from freshly produced *F. graminearum* colonies and placed in the center of Petri dishes containing potato dextrose agar (PDA; Oxoid Ltd., Basingstoke, United Kingdom) with incorporated botanical powders or fungicide. PDA medium without any botanical powder or fungicide served as control. Mycelium was incubated in the dark at 20°C and 80% RH, and the colony diameter was measured when the fastest growing colony had covered approximately 90% of the agar surface. Each treatment included four replicates and the bioassay was conducted twice.

### Growth Chamber Experiment

#### Experimental Design and Procedure

This experiment was conducted in pots in the growth chamber and included three experimental factors (“spraying agent” × “spore type” × “wheat variety”) in a completely randomized design. The spraying agent included Ti, PYM, POM, OMB, a fungicide treatment and the positive control (no antifungal treatment); the spore type included inoculation with either conidia or ascospores; and the wheat variety included the spring wheat varieties Digana and Fiorina (early ripening and medium late ripening, respectively, both with moderate susceptibility to FHB; Delley Seeds and Plants Ltd., Switzerland). Non-inoculated plants (i.e. no infection with conidia or ascospores) served as a negative control. The resulting 26 treatment combinations were replicated four times and the experiment was conducted twice.

Three wheat seeds were sown in 2-liter pots containing potting soil. Prior to sowing, each pot received 6 g of long-term granular fertilizer with micronutrients (N-P-K (MgO) 15-9-12 (2); Standard 5–6 M, Osmocote Exact Standard, Everris, Netherlands). The plants were kept in the growth chamber using the following climatic conditions: seedling growth with 15 h light at 17°C, 75% RH and 9 h dark at 13°C, 85% RH; tillering with 15 h light at 19°C, 70% RH and 9 h dark at 14°C, 80% RH; stem elongation to dough development with 15 h light at 22°C, 70% RH and 9 h dark at 16°C, 80% RH; ripening with 15 h light at 25°C, 70% RH and 9 h dark at 20°C, 80% RH. The light intensity (2:3 Cool White, 1:3 Gro-Lux; Sylvania, Germany) was kept in the range of 350 to 400 μmol m^–2^ s^–1^ throughout the experiment.

The conidial suspensions were prepared by flushing off fresh fungal cultures grown for 5 days at 18°C with a photoperiod 12 h near-ultraviolet light/12 h dark. Perithecia were produced in carrot agar as described in Drakopoulos et al. (2019) with only a modification of the application of Tween^®^ 60 (Sigma-Aldrich, United States) after the removal of the aerial mycelium. Ascospores were flushed off from the lids of the Petri dishes. Both conidia and ascospores were collected using sterile deionized water (dH_2_O) containing 0.0125% Tween^®^ 20.

When plants reached mid- to full anthesis, one inflorescence (head) per plant (i.e. three heads per pot) was labeled before the application of the respective spraying agents and fungal inoculations. The fungicide was sprayed at 0.16% v v^–1^, while botanicals were suspended in sterile dH_2_O at 2% w v^–1^ and stirred for 2 h before application. A blade-type homogenizer (Polytron^®^ PT 3000, Kinematica AG, Switzerland) was used for OMB to reduce the larger particles to smaller fragments. A volume of 20 ml spraying solution was applied to each wheat head using a spray gun (no. 110 nozzle size, 1.5 bar; DeVilbiss PI-7BAR GTi, Carlisle Fluid Technologies, United States). The positive and negative controls were amended with sterile dH_2_O. The following day, each head was inoculated with 2 ml *F. graminearum* spore solution containing 10^4^ conidia or 10^4^ ascospores ml^–1^ and 0.0125% Tween^®^ 20 using a glass vial dispenser (0.5 bar; Sarstedt AG and Co., KG, Germany). The negative control was amended with sterile dH_2_O containing 0.0125% Tween^®^ 20. In order to facilitate infection, plants were transferred to an incubator for 24 h under a continuous fine water mist environment at 20°C in the dark. Subsequently, wheat heads were allowed to dry for 1 h before placing the plants back into the growth chamber.

#### Disease Severity, Yield, Fungal DNA, and Mycotoxins

For the **disease severity**, the number of symptomatic spikelets was counted and expressed as the percentage of infected spikelets per head. The average value from the three labeled heads per pot was calculated. The **grain yield** was measured after manual harvesting and combining the seeds from the three labeled heads per pot. Grain samples were finely ground using a ball mill (frequency of 25 s^–1^ for 60 s; MM400, Retsch GmbH, Germany) and stored at −20°C until further analysis. The ***F. graminearum* DNA in grain** was quantified by CFX96^TM^ Real-Time PCR Detection System for *in vitro* diagnostics (C1000^TM^ Thermal Cycler, Bio-Rad Laboratories, United States) as described for barley in Schöneberg et al. (2018b). In brief, the DNA of 50 mg flour sample was extracted following the protocol of NucleoSpin^®^ 96 Plant II Kit (Macherey-Nagel, Germany) and the amount of total DNA was determined following the Fluorescent DNA Quantitation Kit (BIO-RAD, Switzerland) using a Cary Eclipse Fluorescence Spectrophotometer (Varian, Agilent Technologies, United States) based on the emitted fluorescence of a serially diluted DNA standard. The qPCR method was originally developed by Brandfass and Karlovsky (2006) and adapted according to the available reaction mixtures and laboratory devices. The plasmid contained a 284 bp fragment which is specific to *F. graminearum* (Nicholson et al., 1998). For each qPCR run, samples, standards, and negative control were triplicated. Standards were spiked with DNA from “healthy” wheat flour (wheat variety Apogee cultivated in the greenhouse). The limit of quantification (LOQ) was 40 copies per reaction and the limit of detection one tenth of the LOQ. The **mycotoxins** DON and ZEN in grain were quantified using ELISA kits for enzyme immunoassays (Celer^®^ DON v3 Cod. MD100 and Celer^®^ ZON Cod. MZ670, respectively; Tecna, Italy) with the following modifications: A flour sample of 280 mg was weighed into 2-ml Eppendorf tubes and mycotoxins were extracted with 1.4 ml solvent solution of 70% methanol and 4% sodium chloride (Sigma-Aldrich, United States). Tubes were then vortexed vigorously and shaken horizontally at 250 rpm for 15 min. Samples were centrifuged at 13000 rpm for 5 min and the supernatant was collected. Reference flours with known DON and ZEN contents were extracted the same way (Trilogy Analytical Laboratory, United States). After extraction, ELISA was conducted following the protocol of the kit. The absorbance microplate reader Sunrise^TM^ (TECAN, Austria) was used. When values were below the respective LOQ (DON: LOQ = 0.04 mg kg^–1^; ZEN: LOQ = 0.01 mg kg^–1^), values were replaced by LOQ÷2.

### Field Experiment

#### Experimental Design and Procedure

A field experiment was conducted in 2017 and repeated in 2018 at the research station of Agroscope-Reckenholz in Zurich, Switzerland. A randomized complete block design was followed including two experimental factors (“spraying agent” × “wheat variety”) with four replicates, i.e. square plots of 1 m^2^ each, in blocks. The spraying agents as well as the spring wheat varieties were the same as for the growth chamber experiment. Spring triticale (variety Trado; Saatzucht Düdingen, Switzerland) was cultivated between the wheat plots, serving as buffer plots (1 m^2^ each). To evaluate the performance of the botanicals in field conditions under FHB pressure, a semi-artificial inoculation method was used as described in Drakopoulos et al. (2020) with small modifications. In brief, maize stalks with 30 cm length were autoclaved and then inoculated with a mixture of three *F. graminearum* strains in equal amounts, containing 2 × 10^5^ conidia ml^–1^ and 0.0125% Tween^®^ 20. Ten maize stalks were homogeneously distributed in each wheat plot at seedling growth stage. The preparation, concentrations and equipment for the application of the botanical and fungicide treatments were the same as for the growth chamber experiment. Spraying applications of the botanicals were conducted twice during anthesis of the wheat crop, i.e. at the beginning and at mid-anthesis, while the fungicide was applied at the beginning of anthesis. Sterile dH_2_O served as control. The application rate for all treatments was 600 L ha^–1^.

#### Disease Incidence, Yield, Fungal DNA, and Mycotoxins

For the **disease incidence**, the symptomatic wheat heads were counted per plot. The wheat crop was harvested using a plot combine harvester (Wintersteiger, Austria) and grains were dried for 24 h at 28°C. The seed moisture content (SMC) and hectoliter weight were measured with a moisture tester (GAC 2100, Dickey-John, United States) and **grain yield** was normalized to 12% SMC. For each plot, a representative subsample of 150 g was drawn using a riffle divider (Schiertz and Hauenstein AG, Switzerland) and ground with a mill (Cyclotec^TM^ 1093; Foss Tecator, Sweden) using a 1-mm mesh size. The amount of ***F. graminearum* DNA** and the **mycotoxins** DON and ZEN in grain were quantified as described in the growth chamber experiment, except for the extraction method of mycotoxins due to the higher amount of the harvested grain. Flour samples of 5 g were extracted with 25 ml solvent solution 70% methanol and 4% sodium chloride, shaken for 10 min in an orbital lab shaker at 250 rpm and then passed through folded filters (Whatman^®^, Grade 595 1/2, Sigma-Aldrich, United States).

### Determination of Glucosinolates and Isothiocyanates in Botanicals

Glucosinolate (GSL) extraction from Ti, PYM, POM, and OMB was performed as described in Prestera et al. (1996) with modifications. Twenty g of each botanical powder were weighed into glass tubes and autoclaved at 115°C for 15 min. Then, 1 g of each autoclaved botanical was mixed with 25 ml dH_2_O and homogenized (T18 Ultra-Turrax^®^, Germany) for 3 min at 11000 rpm. The mixture was centrifuged at 4000 rpm for 10 min at 4°C. For the extraction of isothiocyanates (ITCs), non-autoclaved botanical powders were processed following the same method. The obtained extracts were filtered through a 0.22 mm syringe filter and injected (20 μL) into a Shimadzu LC system (Shimadzu, Japan) equipped with a Gemini C18 column (4.6 × 150 mm i.d. 5 mm; Phenomenex, United States). Quantification of GSLs and ITCs was conducted as described in Herzallah and Holley (2012). For the GSL analysis, the mobile phase consisted of 20% (v/v) acetonitrile (ACN) and 80% H_2_O with 0.02 M of tetrabutylammonium hydrogen sulfate (TBA) (final pH 5.5). Detection wavelength was established at 227 nm. For the ITCs determination, the same buffer was used with a different ratio of solvents: 60% ACN (v/v) and 40% H_2_O with 0.02 M TBA. Detection was established at 244 nm. For both analyses of GSLs and ITCs, elution was carried out isocratically for 20 min at a flow rate of 1 ml min^–1^.

### Determination of Phenolic Acids in Botanicals

Phenolic acids were extracted following the Quick, Easy, Cheap, Effective, Rugged, and Safe (QuEChERS) methodology as described in Brosnan et al. (2014). The organic residue obtained by the QuEChERS purification was re-suspended in 1 ml H_2_O:ACN (90:10), passed through a syringe filter, and placed into LC amber vials. Chromatographic determination was performed in an Agilent 1200-LC system (Agilent Technologies, United States) equipped with vacuum degasser, autosampler, and binary pump. The column used for the separation was a Phenomenex Gemini NX-C18 (2 mm Å∼110 mm, particle size 3 μm). Mobile phases consisted of two solvents; solvent A was 0.1% formic acid and solvent B was ACN. The gradient elution program was as follows: at time 0 min, 5% B; then concentration of B increased to 95% until 30 min and kept constant for 5 min; at time 40 min, concentration of B decreased to 5%. Flow rate and injection volume were set at 0.3 ml min^–1^ and 20 μL, respectively. Prior to analysis, the column was equilibrated for 3 min. MS analysis was carried out with 6540 Agilent Ultra-High-Definition Accurate-Mass q-TOF-MS, equipped with Agilent Dual Jet Stream electrospray ionization (Dual AJS ESI) interface in negative ionization mode. The following conditions were established: drying gas was nitrogen at 12 L min^–1^; nebulizer pressure at 50 psi; gas drying temperature at 350°C; capillary voltage at 3500 V; fragmentor voltage at 200 V; scan range at m/z 50-3000. MS/MS analyses were realized with three energy values: 0, 20, and 40 eV. The Mass Hunter Workstation software (Agilent Technologies, United States) was employed for integration and data analysis.

### Statistical Analysis

For the *in vitro* bioassay on mycelium growth, a non-parametric test (Kruskal-Wallis one-way ANOVA) was performed due to largely skewed data and unequal variances across groups. Pooled data from two experiments were used and multiple comparisons among treatments were conducted using the Student-Newman-Keuls method (α = 0.05).

For the growth chamber experiment, a three-way ANOVA was performed to test significance and interactions of the examined factors (“spraying agent” × “spore type” × “wheat variety”) using pooled data from two experiments. To check for normality and homogeneity of variances, data were subjected to Shapiro-Wilk and Brown-Forsythe tests, respectively. In order to approach or reach a normal distribution and homogeneity of variances, a logarithmic transformation was used for the response variables “disease severity,” “*F. graminearum* DNA amount in grain,” and “DON content in grain”. The Duncan’s method was used for the *post hoc* comparisons among treatment groups (α = 0.05).

For the field experiment, a three-way ANOVA was performed to test significance and interactions of the examined factors (“year” × “spraying agent” × “wheat variety”). In order to reach a normal distribution and homogeneity of variances, a square root transformation was used for the response variables “disease incidence”, “*F. graminearum* DNA amount in grain”, and “DON content in grain”. The Duncan’s method was used for the *post hoc* comparisons among treatment groups (α = 0.05). No analysis was performed for the ZEN content in grain for both growth chamber and field experiments, since all measured values were below the LOQ.

A Spearman’s rank-order correlation (*r*_s_) was used to measure the strength and direction of the monotonic relationships between the response variables in the growth chamber and field experiments.

Data were analyzed with the statistical software SigmaPlot 13.0 (Systat Software Inc., United States) and figures were prepared with Prism 5.0 (GraphPad Software Inc., United States). Untransformed data are presented in tables and figures.

## Results

### *In vitro* Bioassay - Mycelium Growth

**FIGURE 1:**
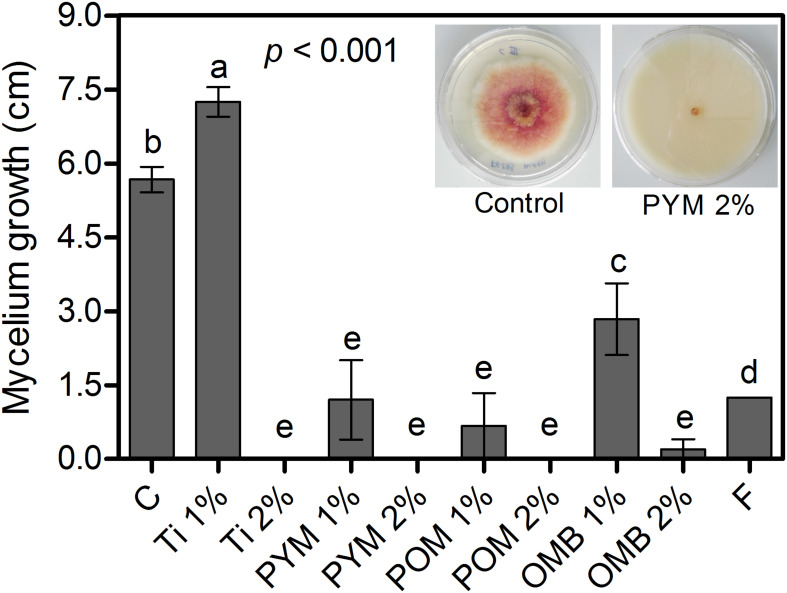
*In vitro* bioassay: Mycelium growth (cm) of *F. graminearum* strain “2113” as affected by Tillecur (Ti), Pure Yellow Mustard (PYM), Pure Oriental Mustard (POM) and Oriental Mustard Bran (OMB) at 1 and 2% concentrations, and Fungicide (F) at 0.16%. C refers to the control treatment. Average values from two experiments are presented and bars represent the standard error of the mean. Different letters indicate significant differences among treatments (α = 0.05).

At 2%, Ti, PYM, and POM completely inhibited mycelium growth of *F. graminearum*, whereas OMB at 2% reduced the growth by 96% compared with the control (Figure 1). At 1%, OMB, PYM, and POM reduced mycelium growth by 50, 79, and 88%, respectively, whereas F reduced the growth by 78% compared with the control (Figure 1).

### Growth Chamber Experiment - Disease Severity, Yield, Fungal DNA, and Mycotoxins

The results from the negative control showed that no cross contamination occurred during the experimental procedure, since no disease symptoms were observed as well as the amount of *F. graminearum* DNA and mycotoxins in grain were below the LOQ. Hence, this treatment was not included in the statistical analysis, and subsequent reference to control only refers to the positive control.

**FIGURE 2:**
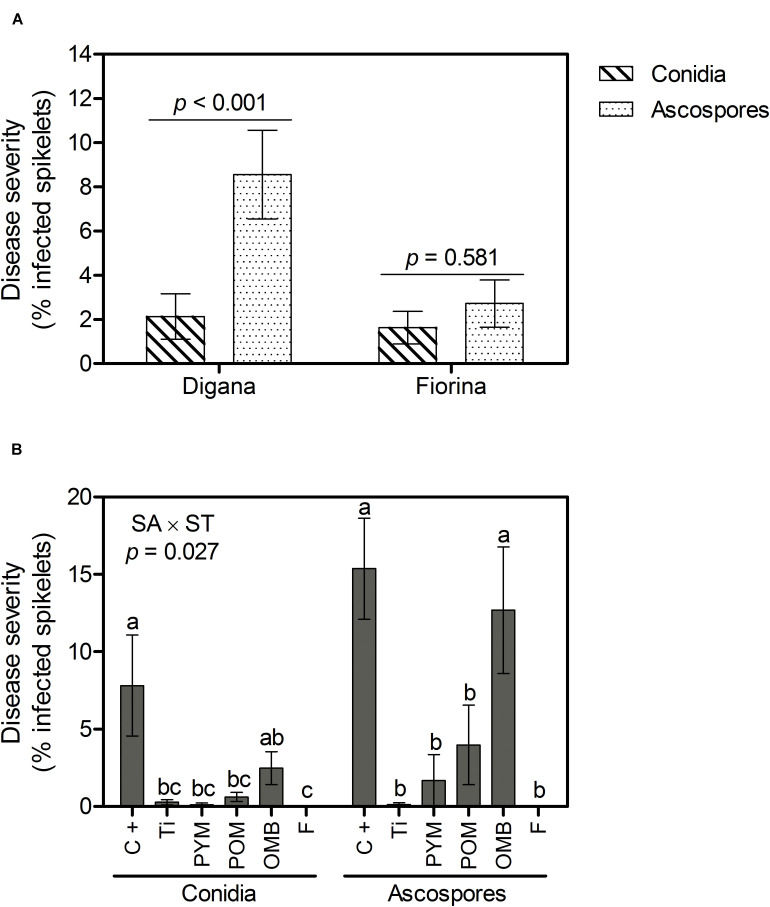
Growth chamber experiment: Disease severity (% infected spikelets) as affected by spore type (conidia, ascospores) within wheat variety (Digana, Fiorina) pooled over the spraying agents **(A)** and as affected by spraying agent (SA) within spore type (ST) pooled over the wheat varieties **(B)**. “SA × ST” stands for the statistical result of the interaction. The used spraying agents were Tillecur (Ti), Pure Yellow Mustard (PYM), Pure Oriental Mustard (POM), Oriental Mustard Bran (OMB), each applied at 2%, and Fungicide (F) at 0.16%. The positive control (C +) refers to infected untreated plants. Average values from two experiments are presented and bars represent the standard error of the mean. Different letters indicate significant differences among treatments (α = 0.05).

Regarding disease severity, significant interactions between spore type and wheat variety (*p* = 0.016) as well as between spraying agent and spore type (*p* = 0.027) were observed (Supplementary Table S1). For Digana, inoculation with ascospores resulted in 4-fold higher disease severity compared with conidia inoculation (*p* < 0.001), while no significant difference was found between conidia and ascospores for Fiorina (*p* = 0.581) (Figure 2A). When inoculation was performed with conidia, all spraying agents resulted in lower disease severity compared with the control, except for OMB (Figure 2B). When inoculation was performed with ascospores, the spraying agents F, Ti, PYM, and POM substantially decreased disease severity compared with the control and OMB (Figure 2B). The disease severity was 2- and 5-fold higher for the control and OMB, respectively, when inoculation was done with ascospores compared with the conidia treatment (Figure 2B).

A significant interaction between spore type and wheat variety (*p* = 0.023) was observed with respect to grain yield (Supplementary Table S1). For Digana, inoculation with ascospores resulted in lower grain yield than with conidia (*p* < 0.001), whereas similar yields were observed between conidial and ascosporic inoculation for Fiorina (*p* > 0.05) (Supplementary Figure S1A). The main effect of spraying agent on grain yield was significant (*p* < 0.001) (Supplementary Table S1). Control and OMB resulted in the lowest grain yield, while F and Ti had the highest, with PYM and POM having intermediate yields (Supplementary Figure S1B).

**FIGURE 3:**
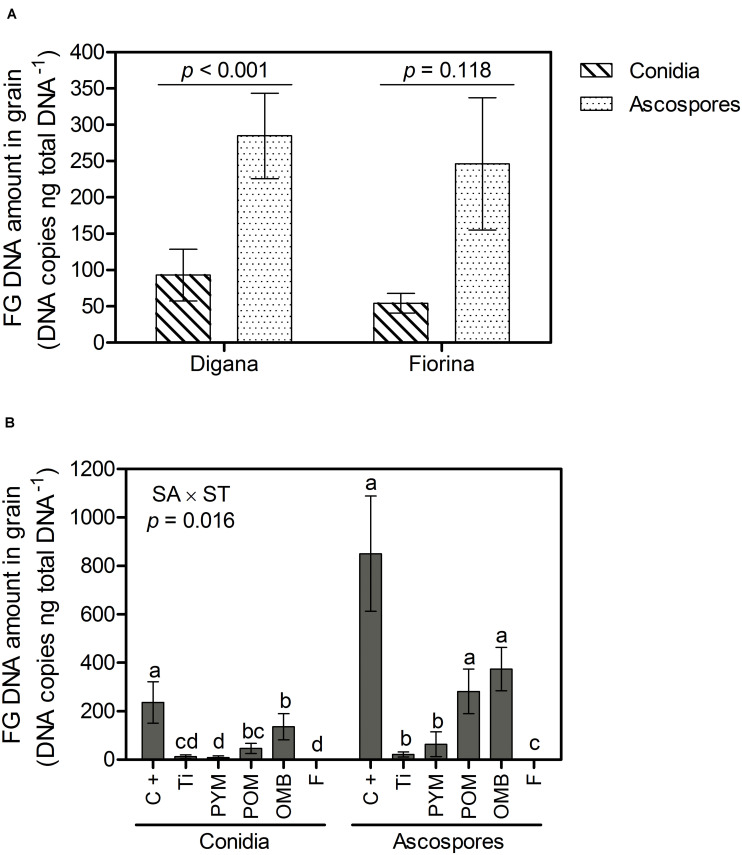
Growth chamber experiment: Amount of *F. graminearum* (FG) DNA in grain (DNA copies ng total DNA^– 1^) as affected by spore type (conidia, ascospores) within wheat variety (Digana, Fiorina) pooled over the spraying agents **(A)** and as affected by spraying agent (SA) within spore type (ST) pooled over the wheat varieties **(B)**. “SA × ST” stands for the statistical result of the interaction. The used spraying agents were Tillecur (Ti), Pure Yellow Mustard (PYM), Pure Oriental Mustard (POM), Oriental Mustard Bran (OMB), each applied at 2%, and Fungicide (F) at 0.16%. The positive control (C+) refers to infected untreated plants. Average values from two experiments are presented and bars represent the standard error of the mean. Different letters indicate significant differences among treatments (α = 0.05).

Regarding *F. graminearum* DNA amount in grain, there was a significant interaction between spore type and wheat variety (*p* = 0.002) as well as between spraying agent and spore type (*p* = 0.016) (Supplementary Table S1). For Digana, inoculation with ascospores resulted in 3-fold higher *F. graminearum* DNA amount in grain compared with conidia, while no significant difference was found between the two spore type inoculations for Fiorina (*p* > 0.05) (Figure 3A). When inoculation was done with conidia, the control led to the highest *F. graminearum* DNA amount, while the spraying agents PYM, Ti, and F led to the lowest, with OMB and POM leading to intermediate DNA amounts (Figure 3B). When inoculation was done with ascospores, the use of F, Ti and PYM resulted in lower amounts of *F. graminearum* DNA compared with the control (Figure 3B). The DNA amount of *F. graminearum* was 8-, 6-, 4-, and 3-fold higher for PYM, POM, control, and OMB treatments, respectively, when inoculation was done with ascospores compared with conidia (Figure 3B).

**FIGURE 4:**
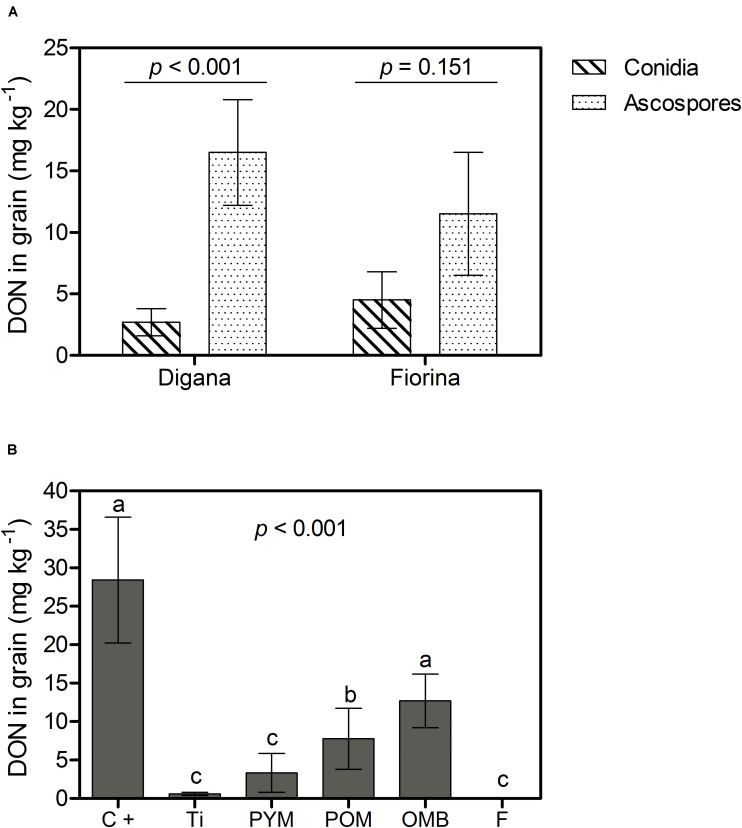
Growth chamber experiment: Deoxynivalenol (DON) content in grain (mg kg^– 1^) as affected by spore type (conidia, ascospores) within wheat variety (Digana, Fiorina) pooled over the spraying agents **(A)** and as affected by spraying agent pooled over the spore types and wheat varieties **(B)**. The used spraying agents were Tillecur (Ti), Pure Yellow Mustard (PYM), Pure Oriental Mustard (POM), Oriental Mustard Bran (OMB), each applied at 2%, and Fungicide (F) at 0.16%. The positive control (C+) refers to infected untreated plants. Average values from two experiments are presented and bars represent the standard error of the mean. Different letters indicate significant differences among treatments (α = 0.05).

As for the previous parameters, there was a significant interaction between spore type and wheat variety (*p* = 0.041) with respect to the DON content in grain (Supplementary Table S1). For Digana, inoculation with ascospores resulted in 6-fold higher DON content compared with conidia (*p* < 0.001), while for Fiorina there was no significant difference between the two spore types (*p* > 0.05) (Figure 4A). The main effect of spraying agent on DON content was significant regardless of spore type or wheat variety (*p* < 0.001) (Supplementary Table S1). DON content was reduced by 100, 98, 88, and 73% with the use of F, Ti, PYM, and POM, respectively, compared with the control, which led to an average DON content of 28.4 mg kg^–1^ (Figure 4B).

Regarding the associations among the examined dependent variables of the growth chamber experiment, positive relationships were observed among disease severity, *F. graminearum* DNA amount, and DON content (*r*_s_ = 0.726 to 0.834, *p* < 0.001, Table 1). Negative correlations were observed between disease severity and grain yield (*r*_s_ = −0.303, *p* < 0.001) as well as between DON content and grain yield (*r*_s_ = −0.307, *p* < 0.001, Table 1).

**TABLE 1:** Growth chamber experiment.

	**Grain yield**	**FG DNA amount**	**DON content**
Disease severity	–0.303	0.726	0.817
Grain yield		–0.238	–0.307
FG DNA amount			0.834

*Spearman’s rank correlation coefficient (*r*_*s*_) indicating the monotonic associations among disease severity, grain yield, *Fusarium graminearum* (FG) DNA amount and deoxynivalenol (DON) content in grain. Data from two experiments were used for the analysis (*n* = 192).*

### Field Experiment – Disease Incidence, Yield, Fungal DNA, and Mycotoxins

**FIGURE 5:**
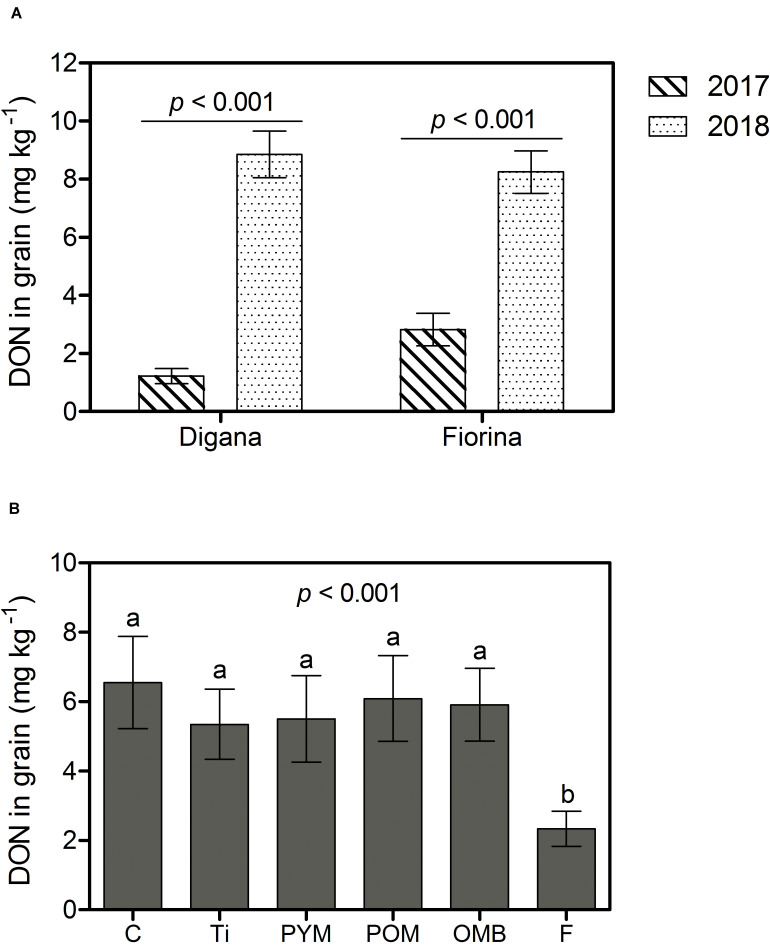
Field experiment: Deoxynivalenol (DON) content in grain (mg kg^– 1^) as affected by year (2017, 2018) within wheat variety (Digana, Fiorina) pooled over the spraying agents **(A)** and as affected by spraying agent pooled over the wheat varieties and the 2 years **(B)**. The used spraying agents were Tillecur (Ti), Pure Yellow Mustard (PYM), Pure Oriental Mustard (POM), Oriental Mustard Bran (OMB) applied at 2%, and Fungicide (F) at 0.16%. Control (C) refers to infected untreated plants. Bars represent the standard error of the mean and different letters indicate significant differences among treatments (α = 0.05).

Overall and for both wheat varieties, the disease incidence, the amount of *F. graminearum* DNA, and the DON content in grain were higher in 2018 than in 2017 (*p* < 0.001) (Supplementary Figures S2A, S3A and Figure 5A). In parallel, grain yield and hectoliter weight were lower in 2018 than in 2017 (*p* < 0.001) (Supplementary Figures S4A, S5A). The main effects of spraying agent on disease incidence (*p* < 0.001), *F. graminearum* DNA amount (*p* = 0.003), DON content (*p* < 0.001), and grain yield (*p* = 0.015) were significant regardless of year or wheat variety (Supplementary Table S2). Compared with the control treatment, the use of F reduced disease incidence by 85%, *F. graminearum* DNA amount in grain by 59%, and DON content in grain by 64%, while none of the botanicals showed significant effects (Supplementary Figures S2B, S3B and Figure 5B). The control, PYM, and POM led to the lowest yields (6.72 to 6.93 t ha^–1^) and F to the highest (7.60 t ha^–1^), while OMB and Ti resulted in intermediate yields (7.11 and 7.16 t ha^–1^, respectively) (Supplementary Figure S4B). Regarding hectoliter weight, there was a significant interaction between year and spraying agent (*p* = 0.005) (Supplementary Table S2). In 2017, there was no difference in hectoliter weight between spraying agents and control, whereas in 2018, the F treatment resulted in higher values compared with all other treatments (Supplementary Figure S5B).

Regarding the associations among the examined dependent variables of the field experiment, positive relationships were observed among disease incidence, *F. graminearum* DNA amount, and DON content (*r*_s_ = 0.869 to 0.973, *p* < 0.001, Table 2). Negative correlations were found between DON content and grain yield (*r*_s_ = −0.732, *p* < 0.001) as well as between DON content and hectoliter weight (*r*_s_ = −0.815, *p* < 0.001, Table 2).

**TABLE 2:** Field experiment.

	**Grain yield**	**Hectoliter weight**	**FG DNA amount**	**DON content**
Disease incidence	–0.670	–0.823	0.869	0.904
Grain yield		0.788	–0.767	–0.732
Hectoliter weight			–0.793	–0.815
FG DNA amount				0.973

*Spearman’s rank correlation coefficient (*r*_*s*_) indicating the monotonic associations among disease incidence, grain yield, hectoliter weight, *Fusarium graminearum* (FG) DNA amount and deoxynivalenol (DON) content in grain. Data from two experiments were used for the analysis (*n* = 96).*

### Determination of Glucosinolates, Isothiocyanates, and Phenolic Acids in Botanicals

The detailed results of GSLs, ITCs as well as those of the phenolic acids identified in the botanical powders Ti, PYM, POM, and OMB are provided in Tables 3, 4. Ti is characterized by an elevated quantity of the GSL sinalbin (56.4 mg g^–1^) which is converted into p-hydroxybenzyl isothiocyanate (p-HBITC) through the myrosinase reaction. The concentration of the latter bioactive compound in Ti was 2.56 mg g^–1^. Sinalbin and p-HBITC were also identified in PYM in similar concentrations, i.e. 57.4 and 2.44 mg g^–1^, respectively. POM and OMB are characterized by the presence of the GSL sinigrin (19.0 and 11.2 mg g^–1^, respectively) which is converted into allyl ITC through the myrosinase reaction. Allyl ITC was detected in POM and OMB matrices at 8.38 and 4.48 mg g^–1^, respectively. Ti contains 14 different phenolic acids in considerable amounts. The compounds with the highest and lowest concentrations were hydroxycinnamic acid (26.1 mg kg^–1^) and gallic acid (0.12 mg kg^–1^), respectively. High concentrations of ferulic acid, benzoic acid, dihydroxybenzene, and phenyllactic acid were detected in this matrix (6.46, 6.35, 2.21, and 2.12 mg kg^–1^, respectively). PYM contains 10 different phenolic acids and the detected compound with the highest concentration in this matrix was benzoic acid (11.8 mg kg^–1^). The other bioactive compounds were detected in a range of concentrations from 0.11 to 0.52 mg kg^–1^. POM contains 10 different phenolic acids at a range of 0.18 to 0.63 mg kg^–1^. Considerable concentrations of p-coumaric, sinapic acid, and vanillic acid were detected in OMB (4.26, 1.26, and 1.01 mg kg^–1^, respectively).

**TABLE 3:** Glucosinolates (GSLs, mg g^–1^) and isothiocyanates (ITCs, mg g^–1^) identified and quantified in Tillecur (Ti), Pure Yellow Mustard (PYM), Pure Oriental Mustard (POM), and Oriental Mustard Bran (OMB).

	**GSLs**		**ITCs**	
**Botanicals**	**Sinigrin**	**Sinalbin**	**Allyl ITC**	**p-HBITC**
Ti	nd	56.44 ± 0.02	nd	2.56 ± 0.08
PYM	nd	57.41 ± 0.12	nd	2.44 ± 0.05
POM	19.01 ± 1.14	nd	8.38 ± 0.40	nd
OMB	11.24 ± 0.74	nd	4.48 ± 0.96	nd

*nd: not detected; ± standard deviation.*

**TABLE 4:** Phenolic acids (mg kg^–1^) identified and quantified in Tillecur (Ti), Pure Yellow Mustard (PYM), Pure Oriental Mustard (POM), and Oriental Mustard Bran (OMB).

**Phenolic acids**	**Ti**	**PYM**	**POM**	**OMB**
1-2-Dihydroxybenzene	2.21 ± 0.01	nd	nd	0.11 ± 0.02
3-4-Dihydroxyhydrocinnamic	0.13 ± 0.01	0.20 ± 0.02	nd	nd
Benzoic acid	6.35 ± 0.04	11.83 ± 0.02	0.63 ± 0.02	0.82 ± 0.01
Caffeic acid	0.21 ± 0.01	nd	nd	0.31 ± 0.01
Chlorogenic acid	nd	nd	0.18 ± 0.10	nd
DL-3-Phenyllactic acid	2.12 ± 0.04	0.34 ± 0.01	0.28 ± 0.01	0.30 ± 0.03
Ferulic acid	6.46 ± 0.02	0.33 ± 0.01	0.37 ± 0.02	0.74 ± 0.04
Gallic acid	0.12 ± 0.02	nd	nd	nd
Hydroxycinnamic acid	26.12 ± 0.01	0.52 ± 0.03	0.56 ± 0.01	0.44 ± 0.02
P-Coumaric acid	0.35 ± 0.03	0.21 ± 0.01	0.55 ± 0.02	4.26 ± 0.03
Protocatechuic acid	1.28 ± 0.02	0.25 ± 0.04	nd	nd
Sinapic acid	0.57 ± 0.02	0.27 ± 0.01	0.62 ± 0.04	1.26 ± 0.03
Syringic acid	0.13 ± 0.01	0.11 ± 0.01	0.43 ± 0.04	0.16 ± 0.02
Vanillic acid	0.48 ± 0.02	nd	0.51 ± 0.01	1.01 ± 0.02
Vanillin	0.29 ± 0.01	0.21 ± 0.02	0.30 ± 0.02	0.14 ± 0.01

*nd: not detected; ± standard deviation*

## Discussion

In this study, the efficacy of mustard-based botanicals against *F. graminearum* infection and mycotoxin accumulation in wheat was investigated under controlled environment and under field conditions. Furthermore, the effect of inoculum on the infection of wheat heads, i.e. with conidia or ascospores, on disease severity and mycotoxin production was studied. In a previous research, the potential of yellow mustard seed flours (Ti, PYM) to suppress or fully inhibit *in vitro* growth and development of *F. graminearum* was already demonstrated (Drakopoulos et al., 2019). In the current study, further botanicals, based on Indian mustard (POM, OMB), and a synthetic prothioconazole fungicide (F) were included.

Remarkably, at 2%, all botanicals showed a higher efficacy in inhibiting mycelium growth *in vitro* compared with F. In the growth chamber experiment, the best performing spraying agents to reduce the DON content in grain were F, Ti, and PYM. Mustard-based botanicals have been extensively studied in the past for their potent antimicrobial activity (Saladino et al., 2017). Clemente et al. (2016) found that allyl ITC, which is derived from the main GSL sinigrin of Indian mustard, had stronger bactericidal and bacteriostatic properties than cinnamon essential oil in broth dilution, direct contact, and vapor phase assays. Luciano and Holley (2009) suggested that allyl ITC has a multi-targeted mechanism of action, damaging cellular structures and inhibiting several metabolic pathways. Allyl ITC reduced the growth of the mycotoxigenic fungi *Aspergillus parasiticus* and *Penicillium expansum* on soil medium (Manyes et al., 2015) and completely inhibited the production of aflatoxins by *A. parasiticus*, as well as beauvericin and enniatin by *Fusarium poae* in wheat flour (Meca et al., 2012; Nazareth et al., 2016). In our study, allyl ITC was detected in both matrices of POM and OMB with 8.38 and 4.48 mg g^–1^ concentrations, respectively. Ekanayake et al. (2006) reported the potential use of white mustard essential oil for food preservation due to the presence of p-HBITC, which is produced by hydrolysis of the GSL sinalbin. David et al. (2013) also showed good efficacy of p-HBITC in controlling *Salmonella* sp. in a frozen sauce with vegetable and chicken particulates. In our study, p-HBITC was detected in both matrices of Ti and PYM with 2.56 and 2.44 mg g^–1^, respectively. Aires et al. (2009), using a disk diffusion *in vitro* assay, showed that ITCs had potent antibacterial activity while the other non-ITC hydrolysis products were much less effective. In addition, the authors indicated that the hydrolysis products from the aromatic group were more effective compared with the aliphatic group. Similarly, the results from the growth chamber experiment showed that Ti and PYM, which contain p-HBITC (aromatic group), had higher efficacy against *F*. *graminearum* infection and DON accumulation in grain compared with POM and OMB, which contain allyl ITC (aliphatic group).

Besides the bioactive ITCs, several antimicrobial phenolic acids were present in the botanical matrices. The concentrations of the detected acids varied substantially among the studied botanicals and corresponded well with the observed higher efficacies of Ti and PYM against *F. graminearum* in the growth chamber experiment. Phenolic acids, such as ferulic and gallic acids, can cause irreversible changes in membrane properties of pathogenic bacteria (Borges et al., 2013). Studies on the mode of action against fungal pathogens are still scarce. Teodoro et al. (2015) summarized some of the described mechanisms of action against *Candida* species including, among others, cell wall damage, disruption of plasma membrane, and inhibition of the isocitrate lyase enzyme activity. Boutigny et al. (2009) found that ferulic acid had a strong inhibitory effect on type B trichothecene biosynthesis in liquid cultures with *F. culmorum*, while Ferrochio et al. (2013) showed that higher doses of ferulic acid reduced the growth rate of *F. verticillioides* and *F. proliferatum* on maize based media. We demonstrated that Ti, which had the highest efficacy in the growth chamber experiment, contained 9- to 20-fold higher concentrations of ferulic acid compared with the other botanicals. In another study, ferulic acid reduced the production of T-2 toxin by *F. langsethiae* and *F. sporotrichioides*, whereas p-coumaric acid stimulated the production of T-2 and HT-2 toxins (Ferruz et al., 2016). This finding might explain our observation that OMB, which contained a higher concentration of p-coumaric acid, showed the lowest efficacy compared with the other spraying agents in the growth chamber experiment. The highest concentrations of benzoic acid were detected in Ti and PYM (6.35 and 11.83 mg kg^–1^, respectively), which are both based on yellow mustard seed flour. Thus, the higher efficacy against *F. graminearum* with the use of Ti and PYM might also be due to the higher amounts of benzoic acid, and its mode of action should be further investigated. Interestingly, Ti contained 50-fold higher concentrations of hydroxycinnamic acid than PYM, which could be the reason for its superior performance in reducing DON content in grain under controlled conditions. Consequently, the antifungal activities of the used botanicals in this study are derived from bioactive matrices containing both ITCs and certain phenolic acids, which could have synergistic effects against *F. graminearum*. Hence, the antifungal effects of individual or combined compounds (i.e. ITCs and phenolic acids) should be tested in future studies.

Under field conditions, only the use of F significantly decreased FHB infection and DON content in grain compared with the control treatment, which stands in contrast to the observed efficacies of the botanicals *in vitro* and *in planta* under controlled conditions. Hence, to improve the efficacy in the field, an effective biopesticide formulation of the mustard-based botanicals is needed. In an earlier study, Ti and PYM were applied on maize residues, which were artificially inoculated with *F. graminearum*, to investigate a potential prevention measure against FHB in the subsequent wheat crop (Drakopoulos et al., 2020). The authors showed that the botanicals were more effective in the second year, compared with the first year, by decreasing DON and ZEN contents in wheat grain up to 41 and 74%, respectively. This lack of consistency in terms of efficacy could be resolved by improving the stability and thus prolonging the activity of ITCs. Allyl ITC in aqueous solution is more unstable under alkaline environments and elevated temperatures (Takatani and Kawakishi, 1995). Liu and Yang (2010) studied the stability and antimicrobial activity of allyl ITC against gram-positive and gram-negative bacteria during long-term storage (180 days) in medium chain triglyceride (MCT) or soybean oil (SBO) dispersed in an oil-in-water (o/w) emulsion system. Higher oil content favored the stability in the emulsion for both MCT and SBO, while, for the same oil content, allyl ITC in MCT was more stable and more effective than in SBO in inhibiting the studied bacteria. Therefore, an effective oil emulsifier may improve the efficacy of the examined mustard-based botanicals under field conditions by stabilizing and prolonging the activity of ITCs. Tracz et al. (2017) evaluated the potential of allyl ITC to inhibit mycotoxin production by *A. parasiticus*, *Alternaria alternata*, *F. tricinctum*, *F. verticillioides*, and *F. graminearum* in maize kernels placed in hermetically sealed flasks. Treatments with allyl ITC kept mycotoxin production below detectable levels, while residual doses (∼16%) of allyl ITC in kernels were detected even after 30 days, indicating an extended protection period. The optimal formulation to improve the stability of mustard-based botanicals should be achieved using environmentally friendly additives with minor impact to the agroecosystem.

Overall, the use of botanicals has several advantages over the use of synthetic pesticides in integrated pest management (Regnault-Roger and Philogène, 2008). For example, botanicals are enzymatically biodegradable with shorter half-lives, since they were biosynthesized. Faster degradation can be an advantage in terms of environmental impact and effects on non-target organisms, but could also decrease the efficacy of the spraying agent as discussed above. Tembo et al. (2018) assessed the potential trade-offs of using plant extracts on legume crop yields and the regulating ecosystem services of natural enemies. Compared with a synthetic insecticide (lambda-cyhalothrin pyrethroid), plant extracts were as effective in terms of crop yields and better conserved the non-target arthropods, suggesting that plant secondary metabolites could be integrated into agroecological crop production systems. Moreover, botanicals belong to several different chemical families and contribute to the diversification of the biochemical and molecular targets towards pests, therefore limiting or delaying the development of resistance (Regnault-Roger and Philogène, 2008). Finally, new pesticide registration procedures, such as the Food Quality Protection Act in the United States, have reduced the availability of synthetic pesticides, leaving more space for the discovery and development of natural product-based pesticides as alternatives in crop protection (Dayan et al., 2009).

An additional remarkable finding of this study is the observed differences between ascosporic and conidial inoculum on *F. graminearum* infection, mycotoxin content, and grain yield under controlled conditions. The disease severity, amount of *F. graminearum* DNA, and DON content were on average higher after inoculating wheat heads with ascospores than conidia, although the results were only significant for the wheat variety Digana. Moreover, inoculation with ascospores resulted in lower grain yield of Digana compared with inoculation with conidia. In fact, for the botanicals OMB, POM, and PYM, the amount of *F. graminearum* DNA in grain was 3- to 8-fold higher following inoculation with ascospores compared with conidia, pointing out that the efficacy of crop protection products depends on the fungal spore type causing the disease. In contrast, the amount of *F. graminearum* DNA in grain was similar after inoculation with ascospores or conidia when plants were treated with Ti or F, which were the most effective spraying agents of this study. This finding suggests that the differences between conidial and ascosporic infections might be eliminated if the efficacy of the product is exceptionally high, which is rare under field conditions. Chinese galls, a botanical rich in gallotannins with antimicrobial activity, inhibited conidia germination, but had minor to no effects on the germination of ascospores (Drakopoulos et al., 2019). This is additional corroboration for our hypothesis that ascospores of *F. graminearum* are more difficult to control than conidia.

The discovery of the different control efficacies towards the two spore types has several potential implications considering that ascospores are more important in causing FHB disease epidemics than conidia. Manstretta et al. (2015) investigated the distribution patterns of ascospores and conidia within a wheat canopy between booting and grain maturity stages using spore traps and found that of the total spores counted, 93% were ascospores and 7% conidia. Mitter et al. (2006) indicated that ascospores were less or equally effective in causing FHB compared with conidia, although differences were small and dependent on wheat type and variety. In addition, Stack (1989) claimed that experimental results based on conidial inoculations can be expected to be valid for ascospores after comparing four inoculum levels, i.e. 1 to 1000 spores per inoculation site. However, these experiments focused on the disease incidence and severity of stem rot in carnation and head blight in durum wheat. An additional explanation for the contrary results could be the genetic variation in the populations of *F. graminearum* resulting in different virulence depending on the fungal strain. The artificial inoculation of inflorescences in studies with FHB causing species is usually performed with conidia. Certainly, conidia are more easily produced than ascospores and the majority of breeders assume that inoculation with conidia might be sufficient to simulate the disease development under field conditions. We suggest that breeding and crop protection programs for resistance and control of *F*. *graminearum* should be conducted using ascospores for the artificial inoculation of the plants or with a mixture of both spore types to better mimic disease epidemics. In the current growth chamber experiment, only one *F. graminearum* strain was used. Therefore, this first evidence should be further supported by investigating the effects of spore type from different fungal strains on disease development and mycotoxin accumulation in several wheat varieties as well as in other small-grain cereals, such as barley and durum wheat.

In conclusion, the efficacy of the mustard-based botanicals Ti, PYM, and POM was excellent under controlled conditions indicating that mustards are highly promising alternatives to synthetic fungicides for FHB control and mycotoxin reduction in wheat. The antifungal activities of the botanicals are derived from the detected ITCs and phenolic acids in the bioactive matrices. However, the dramatically decreased efficacy of the botanicals under field conditions underlines the need for an effective biopesticide formulation. The observed differences between conidial and ascosporic inoculum on disease severity and mycotoxin production suggest that control of *F. graminearum* can be more challenging when infection occurs with ascospores. Finally, in order to improve our understanding of the modes of action, it would be important to isolate the detected ITCs and phenolic acids from the botanical powders and test the antifungal activity of individual or combined compounds against different developmental stages (e.g. mycelium, conidia, and ascospores) of *F. graminearum*.

## Data Availability Statement

The datasets generated for this study are available on request to the corresponding author.

## Author Contributions

SV and DD conceived and designed the experiments. DD was responsible for performing experiments, coordinating the work, and writing the manuscript. GM and RT quantified the glucosinolates, isothiocyanates and phenolic acids in the botanicals, and wrote respective parts of the manuscript. AM, AK, and EJ performed the experiments and measurements. SV, JS, and H-RF provided input with respect to the interpretation of the results and manuscript preparation. All authors contributed to the article and approved the submitted version.

## Conflict of Interest

The authors declare that the research was conducted in the absence of any commercial or financial relationships that could be construed as a potential conflict of interest.
