# Supplementary material for: Control of Fusarium graminearum in Wheat With Mustard-Based Botanicals: From in vitro to in planta
Source: Front Microbiol. 2020 Jul 21;11:1595. doi: 10.3389/fmicb.2020.01595 (PMC7396492; doi:10.3389/fmicb.2020.01595)
Supplement: Supplementary file 1 [file Table_1.DOCX]

# Supplementary Tables and Figures

| **Supplementary Table S1. Growth chamber experiment:** summary of significance levels (*p*-values) from a three-way ANOVA presenting main effects and interactions of spraying agent (SA), spore type (ST) and wheat variety (WV) on disease severity, grain yield, *Fusarium graminearum* (FG) DNA amount and DON content in grain. Data from two experiments were used for the analysis (n = 192). | | | | |
| --- | --- | --- | --- | --- |
| Source of variation | Disease severity | Grain yield | FG DNA amount | DON content |
| SA | < 0.001 | < 0.001 | < 0.001 | < 0.001 |
| ST | 0.002 | 0.005 | < 0.001 | < 0.001 |
| WV | 0.032 | < 0.001 | 0.899 | 0.612 |
| SA × ST | 0.027 | 0.288 | 0.016 | 0.054 |
| SA × WV | 0.196 | > 0.05 | 0.736 | 0.634 |
| ST × WV | 0.016 | 0.023 | 0.002 | 0.041 |
| SA × ST × WV | 0.480 | 0.576 | 0.275 | 0.640 |

| **Supplementary Table S2. Field experiment:** summary of significance levels (*p*-values) from a three-way ANOVA presenting main effects and interactions of year (Y), spraying agent (SA) and wheat variety (WV) on disease incidence, grain yield, hectoliter weight, *Fusarium graminearum* (FG) DNA amount and DON content in grain. Data from two experiments were used for the analysis (n = 96). | | | | | |
| --- | --- | --- | --- | --- | --- |
| Source of variation | Disease incidence | Grain yield | Hectoliter weight | FG DNA amount | DON content |
| Y | < 0.001 | < 0.001 | < 0.001 | < 0.001 | < 0.001 |
| SA | < 0.001 | 0.015 | < 0.001 | 0.003 | < 0.001 |
| WV | 0.230 | 0.027 | 0.014 | 0.076 | 0.157 |
| Y × SA | 0.150 | 0.340 | 0.005 | 0.548 | 0.640 |
| Y × WV | < 0.001 | < 0.001 | < 0.001 | 0.424 | 0.022 |
| SA × WV | 0.474 | 0.567 | 0.292 | 0.336 | 0.261 |
| Y × SA × WV | 0.613 | 0.827 | 0.632 | 0.944 | 0.937 |





**Supplementary Figure S1.** **Growth chamber experiment: grain yield (g pot^-1^)** as affected by spore type (conidia, ascospores) within wheat variety (Digana, Fiorina) pooled over the spraying agents **(A)** and as affected by spraying agent pooled over the wheat varieties and the spore types **(B)**. The used spraying agents were Tillecur (Ti), Pure Yellow Mustard (PYM), Pure Oriental Mustard (POM), Oriental Mustard Bran (OMB) applied at 2 %, and Fungicide (F) at 0.16 %. Positive control (C +) refers to infected untreated plants. Average values from two experiments are presented and bars indicate the standard error of the mean. Different letters indicate significant differences among treatments (α = 0.05).





**Supplementary Figure S2. Field experiment: disease incidence (number of infected heads plot^-1^)** as affected by year (2017, 2018) within wheat variety (Digana, Fiorina) pooled over the spraying agents **(A)** and as affected by spraying agent pooled over the wheat varieties and the two years **(B)**. The used spraying agents were Tillecur (Ti), Pure Yellow Mustard (PYM), Pure Oriental Mustard (POM), Oriental Mustard Bran (OMB) applied at 2 %, and Fungicide (F) at 0.16 %. Control (C) refers to infected untreated plants. Bars represent the standard error of the mean and different letters indicate significant differences among treatments (α = 0.05).





**Supplementary Figure S3.** **Field experiment: amount of *F. graminearum* (FG) DNA in grain (DNA copies ng total DNA^-1^)** as affected by year (2017, 2018) pooled over the wheat varieties (Digana, Fiorina) and the spraying agents **(A)** and as affected by spraying agent pooled over the wheat varieties and the two years **(B)**. The used spraying agents were Tillecur (Ti), Pure Yellow Mustard (PYM), Pure Oriental Mustard (POM), Oriental Mustard Bran (OMB) applied at 2 %, and Fungicide (F) at 0.16 %. Control (C) refers to infected untreated plants. Bars represent the standard error of the mean and different letters indicate significant differences among treatments (α = 0.05).





**Supplementary Figure S4.** **Field experiment: grain yield (t ha^-1^)** as affected by year (2017, 2018) within wheat variety (Digana, Fiorina) pooled over the spraying agents **(A)** and as affected by spraying agent pooled over the wheat varieties and the two years **(B)**. The used spraying agents were Tillecur (Ti), Pure Yellow Mustard (PYM), Pure Oriental Mustard (POM), Oriental Mustard Bran (OMB) applied at 2 %, and Fungicide (F) at 0.16 %. Control (C) refers to infected untreated plants. Bars indicate the standard error of the mean and different letters indicate significant differences among treatments (α = 0.05).

**

**

**Supplementary Figure S5.** **Field experiment: hectoliter weight (kg hl^-1^)** as affected by year (2017, 2018) within wheat variety (Digana, Fiorina) pooled over the spraying agents **(A)** and as affected by spraying agent (SA) within year (Y) pooled over the wheat varieties **(B;** ‘Y × SA’ stands for the statistical result of the interaction**)**. The used spraying agents were Tillecur (Ti), Pure Yellow Mustard (PYM), Pure Oriental Mustard (POM), Oriental Mustard Bran (OMB) applied at 2 %, and Fungicide (F) at 0.16 %. Control (C) refers to infected untreated plants. Bars indicate the standard error of the mean and different letters indicate significant differences among treatments (α = 0.05).


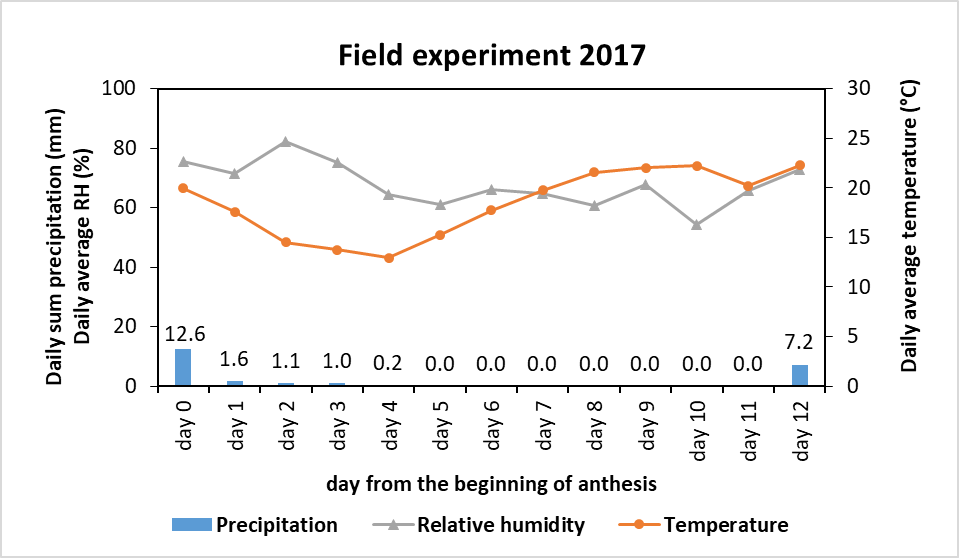


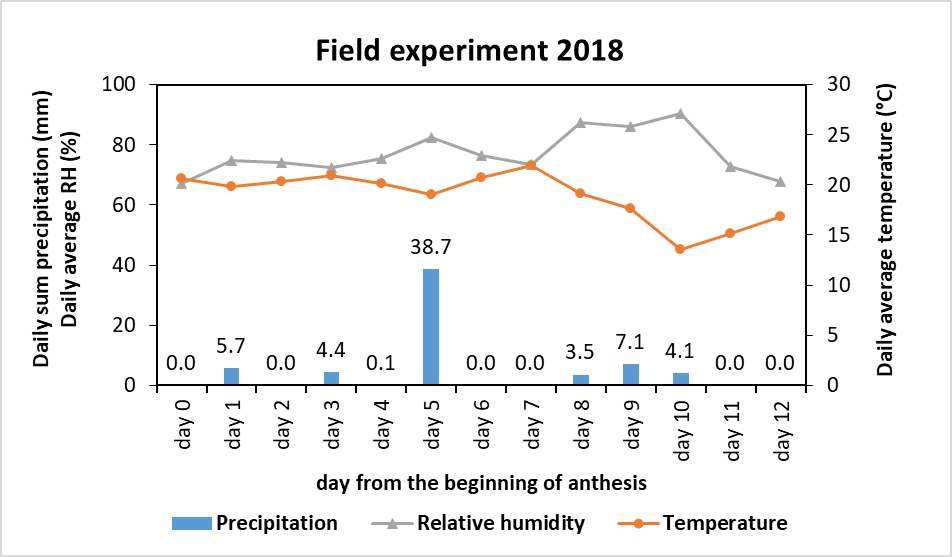


**Supplementary Figure S6.** **Field experiment: climatic data** for the period from the beginning of anthesis (BBCH 61) until the seed watery ripe (BBCH 71) of wheat. The climatic data were obtained from a nearby (< 500 m) weather station (MeteoSwiss, Federal Office of Meteorology and Climatology). Hourly data for temperature (°C), relative humidity (RH %) and precipitation (mm) were retrieved. The average daily values were calculated for temperature and relative humidity and the daily sum for precipitation.
